# Efficacy evaluation of "Dat-e Adolescence": A dating violence prevention program in Spain

**DOI:** 10.1371/journal.pone.0205802

**Published:** 2018-10-15

**Authors:** Virginia Sánchez-Jiménez, Noelia Muñoz-Fernández, Javier Ortega-Rivera

**Affiliations:** Department of Developmental and Educational Psychology, University of Seville, Seville, Spain; Universidade Federal de Sao Paulo, BRAZIL

## Abstract

This study presents the first evaluation of Dat-e Adolescence, a dating violence prevention program aimed at adolescents in Spain. A cluster randomized control trial was used involving two groups (a control group and experimental group) and two waves (pre-test and post-test six months apart). 1,764 students from across seven state high schools in Andalucía (southern Spain) participated in the study (856 in the control group and 908 in the experimental group); 52.3% were boys (*n =* 918), with ages ranging from 11 to 19 years (average age = 14.73; *SD =* 1.34). Efficacy evaluation was analyzed using Latent Change Score Models and showed that the program did not impact on physical, psychological or online aggression and victimization, nor did it modify couple quality. It was, however, effective at modifying myths about romantic love, improving self-esteem, and improving anger regulation, as a trend. These initial results are promising and represent one of the first prevention programs evaluated in this country. Future follow-up will allow us to verify whether these results remain stable in the medium term.

## Introduction

Dating violence, considered a subtype of intimate partner violence, has shown itself to be a construct that poses scientific challenges, its analysis encompassing biological, social, cultural and ideological factors [1]. When we talk about violence in adolescent romantic relationships, we are referring to aggressive behavior, be it verbal, psychological, physical, sexual, or via new technologies, which occurs in relationships that are more or less stable or lasting, current or past [2]. Dating violence is characterized for being primarily contextual, linked to conflicts within the couple [3], and reciprocal [4], with prevalence rates that reach 20% for physical violence, 9% for sexual violence [5], and far higher involvement rates for psychological violence ranging from 20% to 80% [6, 7]. In terms of online violence, and despite being an emerging study phenomenon, data indicate that it occurs between 5% and 56% of cases, depending on the severity of the behavior under analysis [8]. These data, coupled with the serious consequences that violence has on the health and well-being of adolescent populations, such as worse psychological adjustment, drug consumption, suicide attempt, internalizing and/or externalizing problems, among others [9–11], have turned it into a global public health problem [12], which calls for the development of evidence-based intervention programs.

To date, evidence-based programs have mainly been carried out in the United States and Canada; and they are still scarce across Europe and South America [13]. Meta-analyses and systematic reviews conducted thus far [14,15,13] coincide in terms of heterogeneity across different programs, their moderate methodological quality, and their efficacy in bringing about changes in knowledge and beliefs associated with love and violence reaching effect sizes of .47 [14]. However, data on their efficacy in reducing dating aggression and/or victimization have proved less conclusive; only three randomized controlled trials (RCTs) have been found to yield positive outcomes in this area, namely *Safe Dates* [16], *The Fourth R* [17], and S*tepping Stones* [18] although, the effect sizes of these interventions were from moderate to low. Studies revealed that the effects of the programs on dating aggression and victimization seem to be low at post-test, particularly for aggression outcomes (lower than -.19) [17], whereas these effects increase at medium-long term, with effect sizes around -.36 for moderate forms of physical aggression and -.49 for moderate physical victimization [16]. Efficacy in reducing online violence is still unknown territory. In this respect, only two programs have addressed this new form of interpersonal violence, these being *Start Strong* [19] and the adaptation of Safe Dates to an at-risk population, *Moms and Teens for Safe Dates* [20]; and although promising results have been reported for cyber-aggression [20,19], they should be interpreted with caution.

In the case of Spain, research into dating violence is in its infancy [21], yielding prevalence rates for physical violence [22, 23], psychological violence [24], sexual violence [25], and online violence [26] similar to those reported in international studies. The development of evidence-based programs has been limited and, given the variability in approaches, duration, methodological designs, and components, it is difficult to draw conclusions on this subject. Thus, some of these programs have focused on attitudinal changes and changes in knowledge relating to gender-based violence [27, 28], whereas others have incorporated dating violence issues into more extensive programs about sex education [29] and about interpersonal violence in adolescence such as bullying and racism [30]. These programs vary in length, lasting from one session [31] to 14 [32], and in methodological design, this being one of the biggest concerns and the primary obstacle when it comes to evaluating their efficacy [33]. To summarize, none of the programs adopted RCTs; some did not include control groups [34, 32]; and others used very small samples [35], meaning that today we cannot make claims as to the efficacy of dating violence prevention programs in Spain.

Within this framework, the adolescent dating violence prevention program *Dat-e Adolescence* has emerged as a response to the need to develop evidence-based prevention programs in our country.

### Theoretical model of Dat-e Adolescence

The *Dat-e Adolescence* program is based on the Dynamic Developmental Systems Model [36], which allows us to examine adolescent dating violence as a dynamic process that combines three important factors or dimensions analyzed from a life span perspective: *dyad members’ characteristics*, which cover their own developmental history and learning experiences, characteristics related to emotion regulation, cognitive skills, beliefs and attitudes toward violence, self-esteem, through to aggressive self-expression; contextual factors associated with the *family* (coercive parenting practices, family violence, and justifying and acceptance attitudes toward violence) and *peers* (the presence and acceptance of peer group violence); and the *couple*’s own relational dynamic, which would produce and reinforce conflictual relationship styles that would escalate into aggressive behavior. Violence, therefore, would be seen not as an individual process but as the product of interaction within different systems, where the developmental characteristics of both partners would converge in a specific context or situation that would lead to conflict escalating into violence.

By adopting this model, the program was designed to influence some *individual variables* directly related to violence, for example, emotion regulation, self-esteem and beliefs and attitudes; the *couple’s relational dynamic*, including positive and negative dynamics, and conflict resolution strategies; and *peer group influence* on violence and other risk factors, with the aim to reduce physical, psychological and online aggression and victimization.

### Structure and contents of Dat-e Adolescence

The *Dat-e Adolescence* program is a multi-component, school-based prevention program directed at young people between the ages of 12 and 19 years. It comprises seven 1-hour long sessions that can be implemented during school hours. The characteristics that define the program are as follows: a) it addresses traditional and online forms of violence to help boys and girls become aware of the different expressions of violence that dating couples may experience; b) it takes into account that dating violence is mainly mutual or reciprocal [4, 37]; c) it involves intervention-oriented activities that examine the associated risk factors [36], emphasizing the important role that beliefs, attitudes and conflict resolution strategies play in the couple’s relational dynamic; d) following the recommendations of previous meta-analyses [14,15], and the positive outcomes being achieved by these programs in preventing dating violence [38] and bullying [39], the role of peers was emphasized in this program. Specifically, it incorporated a peer model component, meaning that classmates themselves are tasked with leading some of the sessions; e) it combines classroom and web-based activities, the latter via the program’s online platform; f) it welcomes a final activity organized by the participating schools covering the main content and lessons learned following intervention; and g) it adopts a constructivist and experiential approach that encourages content learning through different teaching and learning experiences. The proposed activities include role-playing, watching videos, debates, decision-making games, displays and group dynamic exercises.

An initial pilot study was conducted in 2012; some of the program components were tested in a long-format, 17-session version, which obtained positive outcomes in improved couple quality [35]. However, this pilot study was limited to just one school and featured a small, unrepresentative sample. The *Dat-e Adolescence* program incorporates the learnings obtained from this pilot study. New content and components were added and others removed or adapted. The number of sessions was also scaled back.

This study provides an initial assessment of the efficacy of the *Dat-e Adolescence* program on some of the outcomes identified in the intervention. Specifically, we analyze the program’s efficacy in modifying beliefs about romantic love, the impact on self-esteem and on emotion regulation, the impact on couple quality (positive and negative), and on reducing physical, psychological and online aggression and victimization. According to previous literature, we hypothesized that the program will modify beliefs about romantic love, improve participants’ emotion regulation and enhance participants’ couple quality. In relation to behavioral outcomes, we hypothesized that the program would reduce dating aggression and victimization among those participants in the experimental group in comparison to those in the control group. However, we expected to find stronger effects of the program on beliefs and personal variables than on behavioral outcomes [14,15,13].

## Materials and method

### Study design

A Cluster-randomized control trial was used involving a control group and an experimental group. The unit of randomization was the school. The Regional Education Authority provided a list of 15 centers from Seville and Córdoba (Andalucía Region) selected according to a list of criteria proposed by the researchers: Schools from Seville and Cordoba were selected to ensure that researchers could implement the program. The first criterion proposed by the researchers was that all the schools should present a medium economic, social and cultural level (ISC Index in Spain) in accordance with the ranking established by the autonomous region’s Education Authority. This criterion was used in order to exclude schools with very high and low economic, social and cultural levels because medium schools were more representative of the regional situation. The second criterion was that the schools must be public or partially funded by the Regional Government. The government selected these centers using a simple randomization procedure (a list of random numbers was generated following a computer-based program. Those numbers that coincided with the school identification numbers were picked for the study). Of the 15 schools selected, (nine from Seville and six from Córdoba), five refused to participate in the project because they prioritized other educational programs; three decided to participate in a second edition of the Project; and seven agreed to participate prior to being allocated as control or experimental groups. Four of these centers were from Seville and three from Córdoba. One member of the research group who was not in direct contact with the schools used a coin toss procedure to assign schools to one of the two groups and the third author communicated the results to the schools. Four schools were assigned to the experimental group and the remaining three to the control group. Once allocated to the conditions, and before starting the intervention, two of the experimental group schools purposely selected the classes for the intervention. At least two classes per grade for each school received the intervention (a minimum of eight classes per school). The *Dat-e Adolescence* protocol has been deposited in the protocols.io. The digital object identifier (DOI) link is: dx.doi.org/10.17504/protocols.io.tp8emrw

### Procedure

**Fig 1:**
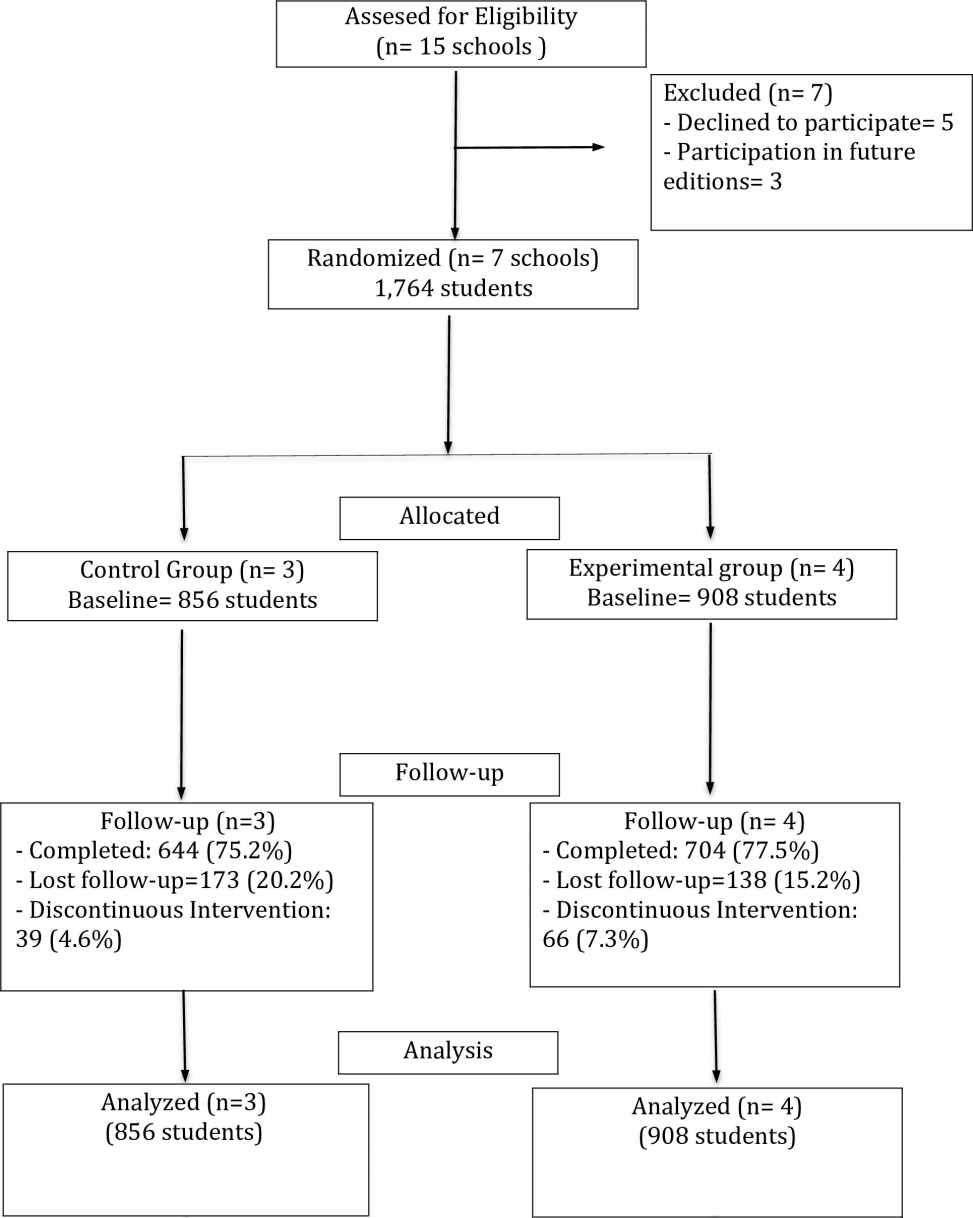
Participant flow. N indicates the number of schools (clusters).

Following approval from the Research Ethics Committee of the Autonomous Region of Andalucía (code: 0575-N-14), contact was initiated with all 15 schools in December 2015. The first author sent an explanation letter to the schools with the aims of the project, the content of the intervention program and the conditions for participation. Once received, the third author (J.O-R) contacted centers by phone and meetings with the heads were established. Meetings were held with the schools’ directors and counseling teams to inform them about the research, its objectives, its content and procedure in the case they were selected as experimental or control groups. Some of the authors attended these meetings. Seven of the 15 schools agreed to participate in the study prior to being allocated as control or experimental group. They signed an informed consent form and forwarded the program details to the families and school boards, the latter granting all centers permission to take part. Once they had the permission, centers were randomly assigned to either an experimental or control group. The waiting list procedure was applied to the control schools that expressed interest in receiving intervention in future editions. The pre-test was carried out in January 2016 and the first post-test in June 2016, around two weeks after the intervention end. The intervention took place from February through May once a week during school hours. The program was implemented by the research staff except for the last two sessions, which were implemented by assistant students with the researchers’ support. In the fifth session two students from each class (one boy and one girl) volunteered to be the implementers of the last two sessions. At each school these assistant students received four hours training (two for each session) prior to the sessions with their peers. On completion of the program, those schools who so desired could conduct a final school activity. No schools took up this option. In sum, researchers visited each class 10 times during the intervention process (see Fig 1).

Anonymous self-report, paper-and-pencil questionnaires were administered to both waves. Data were collected during school hours. Students received no rewards or incentives for taking part.

### Description of participants

#### Sample size

A power analysis was performed to calculate the sample size required to detect a significant effect of the treatment condition on the outcomes. An average intracluster correlation coefficient at school level of ρ = .01 was established according to previous dating violence prevention programs that have reported intraclass correlation coefficients from .006 to .020 [17, 40] depending on the outcomes. In the same way, starting from previous meta-analyses in the area that reported intervention effect sizes from .21 to .47 depending on the outcomes [13, 14, 15],an average effect size of .35 was assumed. A two-tailed test, α = .05 was considered. As a result, 7 clusters and a cluster size of 250 participants were needed to ensure 80% power to detect a significant difference between the experimental and control groups. The research team considered this sample as appropriated taking into consideration that the program was implemented directly by researchers.

#### Participants

1,764 students participated in the study (856 in the control group and 908 in the experimental group); 52.3% were boys (*n =* 918), with ages ranging from 11 to 19 years (average age = 14.73; *SD =* 1.34). 53.1% were in the first two-year cycle of high school education (*n =* 937) and 46.9% in the second two-year cycle (*n =* 827). 1,073 participants studied in the province of Sevilla (60.8%) while 691 studied in the province of Córdoba (39.2%). Regarding romantic experience in wave 1, 470 participants had never dated anyone before (28.9%); 557 had been in a relationship more than two months ago (34.2%); 255 had dated somebody in the last two months (15.7%); and 347 participants were in a current relationship (21.3%). 95.2% identified themselves as heterosexual or straight (*n =* 1673); 1.4% as gay or lesbian (*n =* 24); 1.7% as bisexual (*n =* 30); 0.1% as pansexual (*n =* 1); and 1.6% didn’t know (*n =* 28). Around 96% of participants were born in Spain (*n =* 1687); 2.7% in South America (*n* = 47); 0.8% in Europe (*n* = 14); 0.2% in Asia (*n =* 4); and 0.3% in Africa (*n* = 5). 0.3% of adolescents did not answer this question (*n =* 6). Table 1 provides descriptive data on the experimental and control participants. The two groups were similar in terms of school year, or length of romantic relationship. There were slightly more girls in the experimental group and number of participants with no dating experience was somewhat higher in the experimental group.

**Table 1:** Sample descriptive statistics.

		Control group (*n =* 856)	Experimental group (*n =* 908)	Total (*n =* 1764)
Age; *M (SD)*		14.73 (1.31)	14.75 (1.36)	14.73 (1.34)
Gender; *n* (%)	Girls	422 (49.6%)	496 (54.9%)	836 (47.7%)
	Boys	429 (50.4%)	407 (45.1%)	918 (52.3%)
Education level *n* (%)	1^st^ and 2^nd^ course of Secondary school	473 (55.3%)	464 (51.1%)	937 (53.1%)
	3^rd^ and 4^th^ course of Secondary school	383 (44.7%)	444 (48.9%)	827 (46.9%)
Sentimental experience *n* (%)	No experience	205 (25.4%)	265 (32.2%)	470 (28.9%)
	More than 2 months	281 (34.8%)	276 (33.6%)	557 (34.2%)
	Last two months	136 (16.9%)	119 (14.5%)	255 (15.7%)
	Current relationship	185 (22.9%)	162 (19.7%)	347 (21.3%)
Number of previous romantic relationships; *M (SD)*		3.80 (4.21)	3.29 (3.20)	3.55 (3.76)
Length of current romantic relationship (Number of weeks); *M (SD)*		25.46 (36.15)	24.71 (35.30)	25.09 (35.68)
Length of previous romantic relationship (Number of weeks); *M (SD)*		11.46 (14.99)	8.60 (12.58)	10.09 (13.93)

### Outcomes and measures

#### Sociodemographic variables

An ad hoc questionnaire was devised to ask participants about their gender, age, sexual orientation, locality and nationality.

#### Dating relationship status

Two items from the *Dating questionnaire* [41] were used to analyze relationship status. The first item, a multiple-choice question, assessed the participants’ romantic experience. The response options were as follows: a) Yes, I’m currently dating someone; b) I’m not currently dating anyone, but I have done in the last two months; c) I’m not dating anyone right now but I was more than two months ago; and d) I’ve never dated anyone before. The second item asked about the length of the current or past relationship expressed as number of weeks.

#### Psychological violence

Psychological aggression and victimization were evaluated using the *Psychological Dating Abuse Scale* [42]. Fourteen items, measured on a 5-point Likert scale (0 = Never; 4 = Always), assessed the frequency with which the adolescents, in a current or past relationship, had perpetrated or received abusive behaviors (e.g., “putting the partner down in front of others”, “threatening”, “not letting them do things with other people”, and “blaming the partner for the negative things that he/she has done”, among others). Internal consistency was adequate for both scales in wave 1 and in wave 2: psychological victimization (*α*_*t1*_ = .86; *α*_*t2*_ = .88) and psychological aggression (*α*_*t1*_ = .83; *α*_*t2*_ = .83).

#### Physical violence

Physical aggression and victimization were evaluated using an adapted version of the physical violence scale [43] from the *Conflict Tactics Scale* (CTS2) [44]. Nine items, measured on a 5-point Likert scale (0 = Never; 4 = Always), assessed the frequency with which the adolescents, in a current or past relationship, had perpetrated or received physically violent behaviors (e.g., “pushing”, “slapping”, and “throwing, breaking and kicking things”, among others). Internal consistency was adequate for both scales in wave 1 and in wave 2: physical victimization (*α*_*t1*_ = .82; *α*_*t2*_ = .87) and physical aggression (*α*_*t1*_ = .74; *α*_*t2*_ = .82).

#### Online violence

Online aggression and victimization were evaluated using the non-sexual online violence scale pertaining to the *Cyber Dating Abuse* survey [45]. Nine items, measured on a 5-point Likert scale (0 = Never; 4 = Always), assessed the frequency with which the adolescents perpetrated or received online violent behavior while in a current or past relationship (e.g., “threatening text messages”, “using the partner’s social network account without their permission”, “taking a video of their partner and sending it to other people without their consent”, among others). Internal consistency was adequate for both scales in wave 1 and in wave 2: cyber-victimization (*α*_*t1*_ = .80; *α*_*t2*_ = .80) and cyber-aggression (*α*_*t1*_ = .77; *α*_*t2*_ = .60).

#### Myths about romantic love

An adapted version of the *Myths of Romantic Love Scale* [46] was used. The questionnaire was set at 16 items measured on a 5-point Likert scale, with respondents specifying their level of agreement (0 = Completely disagree; 4 = Completely agree). It was used to analyze a number of romantic myths such as: a) the *myth of omnipotence*, which lies in the belief that love can conquer all (e.g., “If there’s love in a relationship, all problems can be solved”); b) the *myth of jealousy*, which supports the notion that jealousy is a sign of love (e.g., “If your partner is jealous it’s because they truly love you”); c) the *myth of the better half*, which leads us to believe that we are incomplete without the other and that the perfect person is out there for everyone (e.g., “There is someone, somewhere, predestined for each person”); and d) the *myth of eternal passion*, which is the belief that the passion at the start of a relationship should last forever if it’s true love (e.g., “The intense passion of the early stages of a relationship should last forever”). Internal consistency was adequate for all four scales in both wave 1 and wave 2: omnipotence myth (*α*_*t1*_ = .74; *α*_*t2*_ = .79); jealousy myth (*α*_*t1*_ = .86; *α*_*t2*_ = .89); better half myth (*α*_*t1*_ = .70; *α*_*t2*_ = .78); and eternal passion myth (*α*_*t1*_ = .70; *α*_*t2*_ = .81).

#### Couple quality

Negative interactions in adolescent dating relationships and face-to-face (offline) and online intimacy were evaluated. *Negative interactions* were assessed using the conflict scale, criticism scale and antagonism scale taken from the *Network of Relationships Inventory*: *Behavioral Systems Version* [47]. Its nine items, measured on a 5-point Likert scale (0 = Never; 4 = Always), assessed the frequency with which misunderstandings and arguments in a relationship occur (e.g., “How much do you and your romantic partner argue with each other?”, “How much do you and your romantic partner criticize each other?”, “How much do you and your romantic partner get on each other’s nerves?”). *Face-to-face intimacy* was analyzed using the intimacy scale from the *Triangular Love Scale* [48]. Seven items measured on a 7-point Likert scale (0 = Strongly disagree; 6 = Strongly agree) assessed the adolescents’ agreement with sentences related to shared feelings, confidences, ideas and attachment toward their romantic partner (e.g., “I can tell my partner anything”). In addition, the *online intimacy* scale from the *Cyberdating Q-A* instrument [49] was used to analyze intimacy among young people in an online context (e.g., “I always start by greeting my partner affectionately when we connect online”), using three items measured on a 5-point Likert scale (0 = Never; 4 = Always). All the items covering couple quality asked about the current or past relationship. Internal consistency was adequate for all the scales in both wave 1 and wave 2: conflicts (*α*_*t1*_ = .89; *α*_*t2*_ = .91), criticism (*α*_*t1*_ = .80; *α*_*t2*_ = .83), antagonism (*α*_*t1*_ = .76; *α*_*t2*_ = .81); face-to-face intimacy (*α*_*t1*_ = .97; *α*_*t2*_ = .96); and online intimacy (*α*_*t1*_ = .82; *α*_*t2*_ = .81).

#### Anger regulation

An adapted version of the *Emotional Quotient Inventory*: *Youth Version* [50] was used. Eight items measured on a 5-point Likert scale (0 = Never; 4 = Always) assessed difficulties in controlling anger, duration of anger episodes, and the frequency with which adolescents engaged in fights and arguments (e.g., “I find it difficult to control my anger”, “I fight with people”, “When I get angry, I act without thinking”). High scores on the scale suggested less anger regulation and vice versa. Internal consistency was adequate in both wave 1 and wave 2: (*α*_*t1*_ = .83; *α*_*t2*_ = .85).

#### Self-esteem

The *Rosenberg Self-Esteem Scale* [51] was used to measure self-esteem. Ten items analyzed feelings of acceptance toward oneself on a 4-point Likert scale (1 = Strongly disagree; 4 = Strongly agree). Although the scale can be analyzed as a single factor, previous studies have upheld the adequate functioning of two dimensions: *self-confidence* and *self-deprecation* [52, 53]. Internal consistency was adequate for both scales in wave 1 and in wave 2: self-confidence (*α*_*t1*_ = .83; *α*_*t2*_ = .86) and self-deprecation (*α*_*t1*_ = .82; *α*_*t2*_ = .84).

### Attrition analysis

1,348 participants (76.4%) completed the pre-test and post-test measures; 311 adolescents only completed the pre-test (17.6%); and 105 participants only completed the post-test (6%).

The differences in drop-out rates were analyzed according to the condition (experimental vs control) by gender, age, and dating aggression and victimization rates. No differences were observed by gender and age in the control group. However, in the experimental group, participants with missing data were older and more boys than girls only completed the pre-test measure. Despite these differences, the effect size was small (Table 2).

**Table 2:** Analysis of descriptive variables in relation with attrition and experimental condition.

			Participants with pre-test and post-test (*n* = 1348)	Participants only with pre-test (*n* = 311)	Participants only with post-test (*n* = 105)	Diff.
Age *M(SD)*	CG		14.73 (1.32)	14.79 (1.53)	15.07 (1.38)	*F* (2,850) = 1.280; *p* = .279
	EG		14.68 (1.30)	14.82 (1.41)	15.17 (1.23)	*F* (2,893) = 4.235; *p* = .015; *d* = .07
Gender *n* (%)	CG	Boys	312 (48.5%)	91 (53.2%)	19 (51.4%)	*X*^*2*^ *(2) =* 1.239; *p =* .538
		Girls	331 (51.5%)	80 (46.8%)	18 (48.6%)	
	EG	Boys	375 (53.3%)	91 (65.9%)	30 (49.2%)	*X*^*2*^ *(2) =* 8.360; *p =* .015; *C* = .10
		Girls	329 (46.7%)	47 (34.1%)	31 (50.8%)	

*Note*. CG = Control group; EG = Experimental group; diff = comparison among participants’ attrition; F = F-value (one-way); d = Cohen’s d

In the control group, no interaction was observed between attrition and dating violence rates. In contrast, in the experimental group, attrition analysis showed significant differences between participants without attrition and those who only completed the pre-test measure: psychological victimization (*t*(555) = 2.228, *p* = .026, *d* = .19) and psychological aggression (*t* (551) = 2.437, *p* = .015, *d* = .20), although the effect size was small. The average score for psychological victimization and aggression was higher for experimental group participants who only had pre-test (psychological victimization *M* = .40, *SD* = .50; psychological aggression *M* = .35, *SD* = .46) compared to those who showed no attrition (psychological victimization *M* = .29, *SD* = .42; psychological aggression *M* = .25, *SD* = .35). No differences were observed in violence between participants without attrition and those with only post-test.

### Intervention fidelity

In line with the recommendations made by Gottfredson, Cook, Gardner, Gorman-Smith, Howe, Sandler et al. [54], an intervention fidelity analysis was performed aimed at improving its quality and efficacy. An ad hoc, online questionnaire was used for this purpose. Each implementer had to record the following at the end of each session: a) whether they were able to carry out the activities planned for each session (Yes or No answer for each activity); b) the participants’ perceived satisfaction and interest; and c) the implementer’s perception of disruptive behavior during the session. Questions b and c were measured on a 5-point Likert scale (1 = low; 5 = high).

### Plan of analysis

#### Longitudinal measurement invariance

As preliminary analyses, longitudinal measurement invariance [55] was tested for all measures between wave 1 and wave 2 to check that the construct had not changed over time [56]. Several steps were taken to test for measurement invariance: a) *configural invariance*: this is an unconstrained model where the factor structure is the same over time; b) *metric invariance*: the items’ factor loadings are fixed to be equal over time; and c) *scalar invariance*: the factor loadings and intercepts/thresholds are fixed to be equal between wave 1 and wave 2. Evidence of factorial invariance was compared through CFI (ΔCFI) increase between nested models. When the ΔCFI value was above .01 [57], full invariance was rejected, suggesting that some model parameter was not invariant. In these cases, partial measurement invariance was calculated.

#### Latent change score

**Fig 2:**
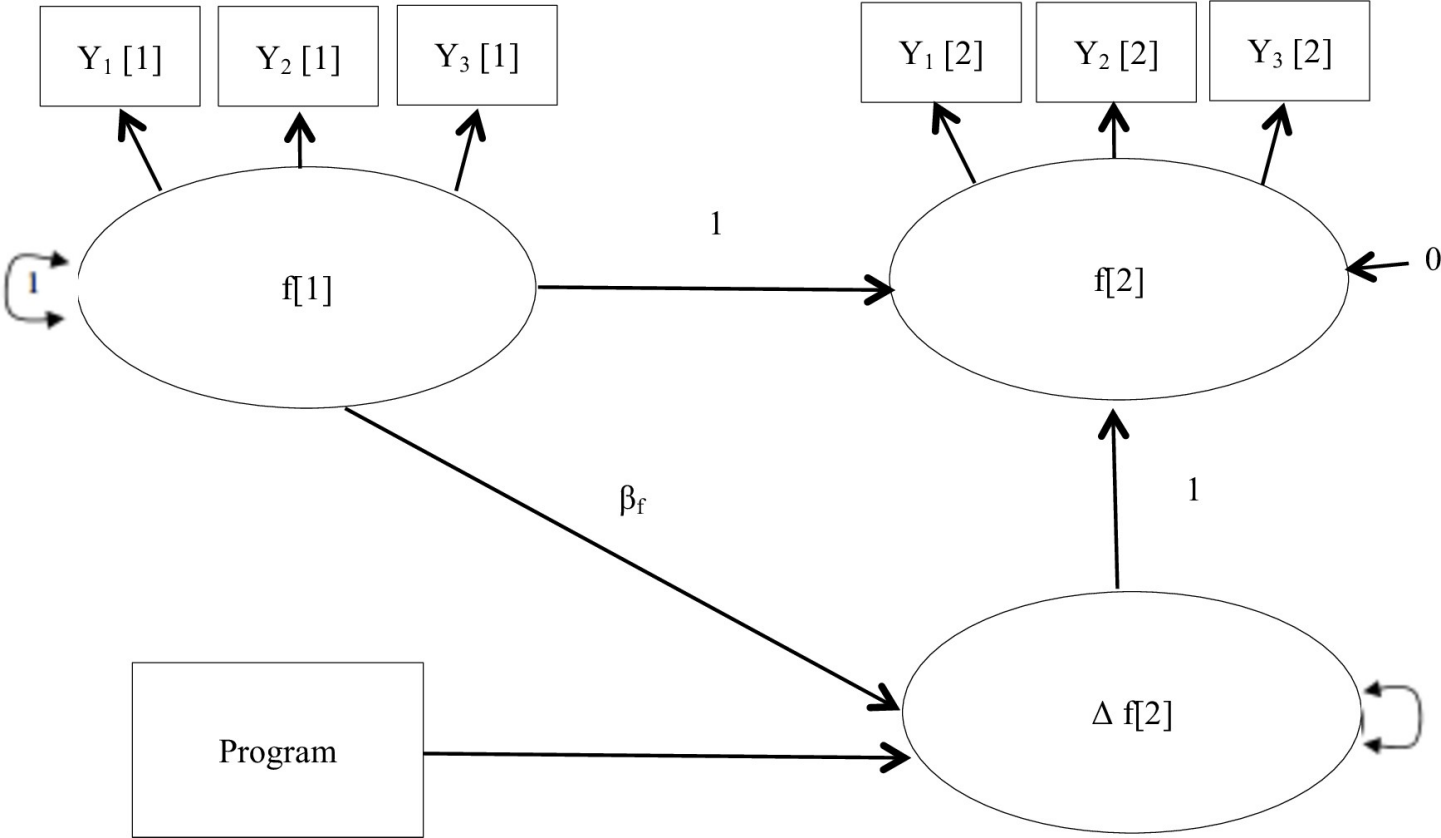
Example of multiple indicator univariate latent change score model and the effect of the program intervention on latent change score. *Note*. Disturbance of f[2] was fixed to 0 to identify the parameters of the model. f[1] and f[2] were measured using multiple indicators. In order to simplify the display of the model, covariance between indicators in wave 1 and wave 2 were not presented.

Latent change score modeling [58] was used to predict longitudinal development and the effect of intervention on the outcomes. This technique is a type of longitudinal growth analysis that makes it possible to analyze the developmental shape of individuals and groups and to test the predictors of these developmental shapes [59]. In line with McArdle [58], the latent difference score (Δ f[2]) is the difference between the subject’s true scores in wave 1 (f[1]) and wave 2 (f[2]), which includes the observed score and the measurement error. Thus, the latent difference score (Δ f[2]) represents the within-subject change across the different observed times. The program’s impact was analyzed by adding its effect to the latent difference score (Fig 2).

All analyses were performed using MPLUS 7 and SPSS 23. The WLSMV estimator [60] was used to analyze dating violence, given that the variables presented normality problems. In the case of physical violence and online violence, the variables were dichotomized due to the low variability of the scales. Psychological violence was also dichotomized in order to facilitate the comparison of the effects of the program on the different outcomes of dating violence. The remaining measures were analyzed using the maximum likelihood (ML) method, except for negative interactions scale, which utilized the maximum likelihood estimation with robust standard errors (MLR). To avoid bias due to sample attrition, all models (except dating violence models) were estimated using the full information maximum likelihood (FIML) method, which makes use of all available information to estimate models with non imputed data. This is the only method that has shown to be successful at avoiding bias by working with missing at random (MAR) data [61]. To perform the dating violence models with WLSMV estimator, we used multiple imputation in MPLUS to generate imputed datasets. We employed the command DATA IMPUTATION to specify the variables for which missing values were imputed (the variables were the items of each outcome with missing values). We imputed five data sets for each outcome. We performed the analysis as many times as outcomes we assessed. Then, we used these new data sets to carry out our models, using TYPE = IMPUTATION to replicate the analysis in all the datasets. The multilevel structure of the data was considered in the inferential analysis using schools as clusters. Intraclass correlations (ICC) for each outcome at student and school level were calculated. This coefficient provides estimates of the proportion of variance due to differences between students and schools. The coefficient effect size was computed by means of the difference between the groups in mean growth rates as the numerator and SD of the slope as the denominator. The following indexes were used to evaluate model fit: the chi-square (X^2^) statistic; the root mean square error of approximation (RMSEA); and the comparative fit index (CFI); with cut-off points of .08 for RMSEA [62] and .90 for CFI [63]. SPSS Statistics 23 software was used for the descriptive analyses as well as for the fidelity and attrition analysis.

## Results

### Preliminary analyses: Longitudinal factorial invariance

Tables 3 and 4 show the results of the longitudinal invariance testing between wave 1 and wave 2 for violence (Table 3) and all other measures (Table 4).

**Table 3:** Models fit for longitudinal factorial invariance and latent change score models for dating violence.

	Longitudinal factorial invariance						Latent change score model		
		*X*^*2*^ *(df)*	*RMSEA*	*CFI*	*ΔCFI*	*Decision*	*X*^*2*^ *(df)*	*RMSEA*	*CFI*
Psychological aggression	Configural	1211.722 (335)	.045	.927					
	Metric	1148.138 (348)	.042	.933	.006	Accepted			
	Scalar	1179.560 (360)	.042	.932	-.001	Accepted	586.318 (388)	.020	.957
Psychological victimization	Configural	1160.064 (335)	.043	.942					
	Metric	1106.643 (348)	.041	.946	.004	Accepted			
	Scalar	1135.767 (361)	.041	.945	-.001	Accepted	610.567 (388)	.021	.964
Physical aggression	Configural^a^	90.411 (69)	.015	.993					
	Metric	92.365 (75)	.013	.994	.001	Accepted			
	Scalar	103.786 (81)	.015	.992	-.002	Accepted	114.763 (94)	.013	.992
Physical victimization	Configural^b^	224.504 (95)	.032	.975					
	Metric	227.044 (102)	.031	.976	.001	Accepted			
	Scalar	235.464 (109)	.030	.976	.000	Accepted	182.624 (124)	.019	.984
Cyber-aggression	Configural	186.740 (125)	.019	.922					
	Metric	196.934 (133)	.019	.919	-.003	Accepted			
	Scalar	204.514 (141)	.019	.919	.000	Accepted	172.945 (158)	.008	.973
Cyber-victimization	Configural^c^	104.274 (95)	.008	.995					
	Metric	116.398 (102)	.010	.992	-.003	Accepted			
	Scalar	127.132 (109)	.011	.990	-.002	Accepted	133.916 (124)	.008	.993

Note.

^a^ item 7 (Slamming or holding against a wall) and 9 (“Choking, punching, or beating”) were deleted of the final scale given that both items showed standardized factors loadings higher than 1.

^b^ item 9 (“Choking, punching, or beating”) was deleted of the final scale given that it showed a standardized factor loading higher than 1.

^c^ item 11 (Created a profile page (like Facebook, Myspace, or YouTube) about me knowing it would upset me) in wave 1 showed a standardized correlation with item 11 in wave 2 higher than 1, for this reason item 11 was deleted of the final scale.

**Table 4:** Models fit for longitudinal factorial invariance and latent change score models for myths of love, quality of relationship, anger management and self-esteem.

		Longitudinal factorial invariance						Latent change Score		
			*X*^*2*^ *(df)*	*RMSEA*	*CFI*	*ΔCFI*	*Decision*	*X*^*2*^ *(df)*	*RMSEA*	*CFI*
Myths of romantic love	Jealousy	Configural	48.788 (15)	.036	.996					
		Metric	59.370 (18)	.036	.995	-.001	Accepted			
		Scalar	67.103 (21)	.035	.994	-.001	Accepted	57.569 (28)	.025	.997
	Better half	Configural	92.432 (15)	.054	.980					
		Metric	105.406 (18)	.053	.977	-.003	Accepted			
		Scalar	114.138 (21)	.050	.975	-.002	Accepted	117.963 (28)	.043	.995
	Omnipotence	Configural	18.492 (15)	.012	.999					
		Metric	19.855 (18)	.008	1.000	.001	Accepted			
		Scalar	39.934 (21)	.023	.995	-.005	Accepted	56.402 (28)	.024	.993
	Passion	Configural^a^	7.365 (5)	.017	.999					
		Metric	14.937 (7)	.025	.997	-.002	Accepted			
		Scalar^b^	49.849 (9)	.051	.985	-.012	Non accepted			
		Partial Scalar	26.737 (8)	.037	.993	-.005	Accepted	56.792 (13)	.044	.985
Quality of romantic relationship	Negative interaction scale (second-order factor)	Configural	337.734 (112)	.039	.971					
		Metric	351.420 (117)	.039	.970	-.001	Accepted			
		Scalar	365.564 (123)	.039	.969	-.001	Accepted	329.571 (144)	.031	.978
	Intimacy f2f	Configural	395.453 (69)	.060	.950					
		Metric	397.579 (75)	.057	.950	.000	Accepted			
		Scalar	422.654 (81)	.057	.947	-.003	Accepted	336.546 (94)	.044	.949
	Intimacy online	Configural	4.193 (5)	.000	1.000					
		Metric	4.828 (7)	.000	1.000	.000	Accepted			
		Scalar	13.767 (9)	.000	.999	-.001	Accepted	18.678 (14)	.016	.998
Psychological variables	Anger regulation	Configural	409.411 (95)	.044	.967					
		Metric	414.748 (102)	.042	.968	.001	Accepted			
		Scalar	426.266 (109)	.041	.967	-.001	Accepted	321.654 (124)	.030	.975
	Self-esteem (2 correlated factors)	Configural	664.719 (154)	.044	.962					
		Metric	670.927 (162)	.043	.962	.000	Accepted			
		Scalar	699.651 (170)	.042	.961	-.001	Accepted	597.747 (190)	.035	.959

Note.

^a^ Item 5 (In a relationship, the passion of the beginning should not diminish over time) showed a high error covariance with item 2 suggesting a high overlapping between the content of both items. In order to get a more parsimonious model, item 5 was not included.

^b^ according to modification index the intercept of item 9 (If a couple really loves each other, over time the passion remains as the first day) was released

As can be observed in Table 3, all models provided an adequate fit and the full scalar invariance level was accepted for both psychological, physical and online aggression and victimization. Regarding physical violence, two items corresponding to the most serious forms of violence were removed; as a result, the items in the final scale mostly reflected expressions of moderate physical violence.

For the remaining variables (Table 4), a good fit was observed across all models accepting the full scalar invariance level, with the exception of the myth of eternal passion, which achieved partial scalar invariance after freeing the intercept for an item in the scale.

Based on these results, it was possible to compare the changes between wave 1 and wave 2 for dating violence, myths about romantic love, couple quality, anger regulation and self-esteem.

### Descriptive analyses

Table 5 shows the mean scores and prevalence rates of the experimental and control groups at pre-test and post-test for violence. At baseline, participants from the control and experimental groups were more involved in psychological aggression and victimization than in the other forms of violence (physical and cyber-aggression). In this respect, 7 out of 10 participants were involved in psychological violence in comparison to around 2 out of 10 who reported perpetrating or receiving physical or online violence.

**Table 5:** Results of dating violence for control group and experimental group in both waves.

	Pre-intervention				Post-intervention			
	Control group		Experimental group		Control group		Experimental group	
	Involved (*%*)	*Mean (SD)*	Involved (*%*)	*Mean (SD)*	Involved (*%*)	*Mean (SD)*	Involved (*%*)	*Mean (SD)*
Psychological aggression	*N =* 589; 431 (73.2%)	.32 (.39)	*N =* 553; 385 (73%)	.27 (.38)	*N =* 490; 336 (68.6%)	.29 (.41)	*N =* 522; 344 (65.9%)	.26 (.38)
Psychological victimization	*N =* 591; 443 (75%)	.35 (.47)	*N =* 557; 394 (71%)	.31 (.44)	*N =* 491; 324 (66%)	.33 (.48)	*N =* 522; 359 (68.8%)	.30 (.45)
Physical aggression	*N =* 588; 91 (15.5%)	.06 (.18)	*N =* 553; 73 (13.2%)	.06 (.22)	*N =* 487; 53 (10.9%)	.05 (.23)	*N =* 520; 55 (10.6%)	.04 (.17)
Physical victimization	*N =* 590; 96 (16.3%)	.06 (.23)	*N =* 556; 87 (15.3%)	.07 (.24)	*N =* 491; 73 (14.9%)	.06 (.24)	*N =* 520; 61 (11.7%)	.05 (.24)
Cyber-aggression	*N =* 592; 81 (13.7%)	.03 (.10)	*N =* 554; 93 (16.5%)	.05 (.19)	*N =* 495; 56 (11.3%)	.03 (.10)	*N =* 530; 83 (15.7%)	.04 (.12)
Cyber-victimization	*N =* 596; 85 (14.3%)	.05 (.18)	*N =* 565; 96 (17%)	.06 (.24)	*N =* 498; 72 (14.5%)	.05 (.20)	*N =* 522; 95 (18%)	.06 (.22)

*Note*. Items about dating violence were answered only for participants with sentimental experience. Differences of total number of subjects (*N*) within the same time are due to missing data. Means and standard deviations are based on subjects with full information available used in the analysis

Table 6 shows the descriptive analyses of the experimental and control groups for myths about romantic love, couple quality, anger regulation and self-esteem in wave 1 and in wave 2. At pre-test, participants presented medium scores in the acceptance of the myths of romantic love, low scores on negative couple quality scales (such as conflicts or criticism) and high levels in positive scales (such as intimacy). In relation to psychological variables, participants showed medium levels in anger regulation as well as for self-esteem scales.

**Table 6:** Results of myths of love, quality of relationship, anger management and self-esteem for control group and experimental group in both waves.

		Pre-intervention		Post-intervention	
		Control group	Experimental group	Control group	Experimental group
		*Mean (SD)*	*Mean (SD)*	*Mean (SD)*	*Mean (SD)*
Myths of romantic love	Jealousy	*N =* 800; 1.60 (1.18)	*N =* 818; 1.56 (1.19)	*N =* 672; 1.38 (1.16)	*N = 764;* .94 (1.04)
	Better half	*N =* 801; 2.14 (1.01)	*N =* 822; 2.14 (1.03)	*N =* 672; 1.98 (1.05)	*N =* 764; 1.42 (1.05)
	Omnipotence	*N =* 801; 2.70 (.98)	*N =* 821; 2.48 (1.01)**	*N =* 672; 2.59 (.99)	*N =* 764; 1.84 (1.07)
	Passion	*N =* 801; 2.83 (.96)	*N =* 821; 2.64 (1.02)**	*N =* 672; 2.77 (.99)	*N =* 764; 1.92 (1.14)
Quality of romantic relationship	Conflicts	*N =* 597; 1.40 (.97)	*N =* 565; 1.21 (.91)**	*N =* 498; 1.44 (.93)	*N =* 531; 1.31 (.96)
	Criticism	*N =* 597; .52 (.71)	*N =* 565; .41 (.66)**	*N =* 498; .56 (.74)	*N =* 530; .51 (.71)
	Antagonism	*N =* 596; 1.06 (.91)	*N =* 563; .95 (.82)**	*N =* 496; 1.09 (.88)	*N =* 529; .99 (.91)
	Intimacy f2f	*N =* 594; 4.05 (1.34)	*N =* 563; 3.92 (1.30)	*N =* 496; 4.16 (1.25)	*N =* 531; 4.00 (1.27)
	Intimacy online	*N =* 595; 2.82 (1.16)	*N =* 564; 2.77 (1.13)	*N =* 498; 2.80 (1.16)	*N =* 529; 2.63 (1.22)
Psychological variables	Anger regulation	*N =* 793; 2.31 (.81)	*N =* 810; 2.24 (.79)	*N =* 668; 2.37 (.85)	*N =* 757; 2.21 (.80)
	Self-confidence	*N =* 773; 3.19 (.63)	*N =* 807; 3.19 (.63)	*N =* 664; 3.15 (.68)	*N =* 754; 3.16 (.66)
	Self-deprecation	*N =* 772; 2.01 (.83)	*N =* 806; 2.00 (.83)	*N =* 664; 2.07 (.88)	*N =* 753; 1.95 (.82)

*Note*. Items about quality of romantic relationship were answered only for participants with sentimental experience. Means and standard deviations are based on subjects with full information available used in the analysis

### Program efficacy on dating violence

A multiple indicator univariate latent change score model was estimated for psychological, physical and online aggression and victimization aimed at analyzing the effect of the *Dat-e Adolescence* program on participant change for each measure after intervention. As can be observed in Table 3, all models yielded a good fit. Regarding the program’s efficacy on violence (Table 7), observations at a descriptive level showed a reduced likelihood of engaging in psychological aggression and victimization, a reduced likelihood of engaging in physical victimization, and an increased likelihood of engaging in physical aggression as well as in cyber-aggression and cyber-victimization following intervention in the experimental group compared with the control group. However, the change observed was not significant.

**Table 7:** Effects of the intervention on outcomes.

		*B*	*SE*	*b*	*p*
Dating violence	LCS -Psychological aggression	-.030	.054	-.035	.576
	LCS -Psychological victimization	-.030	.030	-.029	.317
	LCS -Physical aggression	.026	.083	.025	.753
	LCS -Physical victimization	-.009	.123	.000	.944
	LCS -Cyber-aggression	.120	.105	.091	.253
	LCS -Cybervictimization	.105	.089	.099	.237
Myth of romantic love	LCS -Jealousy	-.448	.076	-.238	.000
	LCS -Better half	-.600	.098	-.359	.000
	LCS -Omnipotence	-.381	.043	-.359	.000
	LCS -Passion	-.598	.065	-.390	.000
Quality of romantic relationship	LCS -Conflicts	-.016	.035	-.010	.658
	LCS -Criticism	.015	.027	.012	.565
	LCS -Antagonism	-.027	.037	-.023	.467
	LCS -Intimacy f2f	-.113	.072	-.054	.118
	LCS -Intimacy online	-.127	.074	-.060	.086
Psychological variables	LCS -Anger regulation	-.110	.062	-.094	.077
	LCS -Self-confidence	-.022	.030	-.025	.494
	LCS -Self-deprecation	-.090	.028	-.070	.001

*Note*. LCS = Latent change score; *B* = beta coefficient; *SE* = standard error; b = standardized beta coefficient; *p* = p-value. ICC estimates: Psychological aggression (ICC-student level = .411; ICC-school level = .002); Psychological victimization (ICC-student level = .359; ICC-school level = .001); Physical aggression (ICC-student level = .243; ICC-school level = .005); Physical victimization (ICC-student level = .270; ICC-school level = .005); Cyber-aggression (ICC-student level = .295; ICC-school level = .003); Cyber-victimization (ICC-student level = .349; ICC-school level = .004); Myth of jealousy (ICC-student level = .525; ICC-school level = .025); Myth of better half (ICC-student level = .417; ICC-school level = .030); Myth of omnipotence (ICC-student level = .376; ICC-school level = .060); Myth of passion (ICC-student level = .281; ICC-school level = .058); Conflicts (ICC-student level = .558; ICC-school level = .008); Criticism (ICC-student level = .442; ICC-school level = .006); Antagonism (ICC-student level = .542; ICC-school level = .006); Intimacy f2f (ICC-student level = .585; ICC-school level = .004); Intimacy online (ICC-student level = .445; ICC-school level = .015); Anger regulation (ICC-student level = .681; ICC-school level = .007); Self-confidence (ICC-student level = .627; ICC-school level = .005); Self-deprecation (ICC-student level = .626; ICC-school level = .002).

The program’s efficacy on psychological aggression and victimization was also estimated considering only those participants without attrition, given the differences found for psychological aggression and victimization between experimental group participants who only took pre-test and those without attrition. The effect of the program for psychological aggression and victimization in the without-attrition sample was similar to that observed including all participants. These results are not reported in the present study, but can be made available upon request from the first author.

### Program efficacy on myths about romantic love

The latent change score was estimated for the four myths of romantic love under analysis (Table 4). The models yielded a good fit in all cases. As expected, the program had a significant impact on change across all myths of romantic love, reporting a reduction in the acceptance of myths among those participants who took the program compared with the control group participants (Table 7). The effect sizes were large for better half myth (*d = -*.*83)*, omnipotence (*d = -*.*84)*, passion, (*d = -*.*94)* and medium for jealousy (*d = -*.*56)*.

### Program efficacy on couple quality

The effect of the program on negative interactions scales (conflicts, criticism, antagonism), face-to-face intimacy and online intimacy were analyzed (Table 4). The fit of the latent change score models was good for all the variables. As shown in Table 7, a significant program effect on change was not observed for negative interactions scales nor for both intimacy measures.

### Program efficacy on anger regulation and self-esteem

Lastly, program efficacy on anger regulation and self-esteem was also analyzed (Table 4). As can be seen in Table 4, the latent change score models yielded a good fit. In terms of anger regulation, a trend was observed. Those participants who received the intervention increased their confidence in the self-regulation of their own anger in comparison to the control group, although the effect size was small (*d = -*.*19)*. In relation to self-esteem, the efficacy results (Table 7) showed that the program had a significant impact on self-deprecation, but not for self-confidence. For self-deprecation, the experimental group participants reduced their scores compared with the change observed in the control group participants but the effect size was small (*d = -*.*15)*.

### Program fidelity analysis

Design fidelity was very high. In the first four sessions, around 85% of the designed content was implemented, reaching 100% in the last three sessions.

Perceived satisfaction was high (average values between 3.94 and 4.60) and disruptive behavior during session implementation was low to moderate across all sessions (average values between 1.47 and 2.69).

## Discussion

This study presents and evaluates the efficacy of the first edition of the *Dat-e Adolescence* program in accordance with the standards of evidence proposed by the Society for Prevention Research [54]. Specifically, the aim of this study was to evaluate the program’s efficacy in reducing adolescent partner aggression and victimization; in regulating anger, self-esteem and beliefs about love and violence; and in relation to some variables associated with couple quality among Spanish adolescents aged 12 to 19 years attending state high schools with medium economic, social and cultural level in the Andalucía region.

The assessment of the program’s efficacy on physical, psychological and online aggression and victimization did not yield the expected results. The latent change score models showed that although a decrease in frequency and involvement of experimental group adolescents for psychological aggression and victimization took place, the change was not sufficient to detect differences among groups. These results are consistent with previously developed prevention programs [38] [64–67] and with the conclusions drawn in the meta-analyses [14, 15], reflecting how resistant to change these violent behaviors can be when they become embedded in couple relationships [68], especially in programs that do not last long enough to allow for the learning and consolidation of healthy coping skills and strategies to tackle violence [14]. Incorporating follow-up measures and booster activities, like those covered in the *Safe Dates* program [69], would not only allow us to test potential program efficacy in the medium-long term, but would also enable us to ascertain whether the consolidation of certain positive conflict resolution skills could lead to violence reduction. Furthermore, the program failed to impact on less frequents forms of violence, such as physical and cyber aggression and victimization despite the data showing a positive trend, which suggests that experimental group participants reported greater involvement frequency in both measures relative to those in the control group. Although subsequent program follow-up measures would confirm whether this trend holds over time [38], this outcome could also indicate young people’s increased awareness and sensitivity toward these forms of violence, particularly online violence given the potentially novel and changing nature of violent phenomena that emerge via new technologies.

On the other hand, “floor effects” of behavioral outcomes could explain why no differences among groups were found, either for aggression or for victimization outcomes. This "floor effect" has been reported in community samples studies about interpersonal aggression, concluding that this effect attenuates the detection of intervention effects of universal programs [13, 18]. In order to avoid this effect, some authors have focused on adolescents with higher baseline exposure to dating violence, comparing the results with those not involved or presenting lower levels at baseline [16]. Because we were assessing the first edition of the program, which was designed to be a universal prevention program, we decided to test its efficacy in the entire sample in a first step. Future evaluations will allow us to test the program efficacy on specific sub-populations, such as participants with higher levels of exposure to violence, or high-risk populations.

Positive outcomes were not found for couple quality. The program failed to improve positive quality, nor did it reduce negative interactions in the experimental group compared to the control group. These findings are consistent with previous studies regarding the non-decrease in negative interactions among couples [19], although they do not coincide with regard to the absence of positive quality improvement. There are several reasons that might explain these results. On the one hand, negative interactions are part of the couple dynamic in the adolescent years, so it would have been interesting to incorporate conflict resolution measures to analyze how adolescents resolve their problems with their partners rather than the frequency with which they occur. In this sense, the measure used does not inform whether the program has improved conflict resolution skills among adolescent couples, only that such negative interactions have not diminished. On the other hand, these findings should also be analyzed in light of the program’s design, given that training-related content focused on conflict-resolution skills was covered in one and a half sessions. Increasing this program content would give participants the chance to consolidate these skills [14]. Lastly, the participants’ baseline levels for dating intimacy were very high, meaning that score variability was too low to be sensitive to change following intervention. This result is in line with previous cross-cultural studies and may reflect a cultural feature of Spanish adolescent couples, since Spanish adolescents presented higher levels of positive quality compared with their Italian [4] and British peers [23]. To include different indicators of positive dating quality, such as relational satisfaction, would have offered a more comprehensive and perhaps sensitive picture of positive couple quality.

The fact that no statistical differences were found in the control and experimental groups either for behavioral outcomes or for couple quality measures could also been explained in terms of the sample size. Our sample was estimated to find effect sizes of around .35 with an 80% of chance of rejecting the hypothesis that both groups were equal. Although the sample estimation was acceptable, the size meant that it was not possible to find small effects, as those found for behavioral outcomes at post-test in previous works [13,14,15]. It could be hypothesized that increasing the sample size to reach a 95% level, could improve the probability of reaching the minimum effect sizes that allow us to make more accurate conclusions about the program efficacy. Future trials could test the program in larger samples.

The *Dat-e Adolescence* program did, however, have a clear effect on beliefs and myths about romantic love, and self-esteem. The results showed that the experimental group participants significantly reduced their degree of agreement with the jealousy, omnipotence, better half and eternal passion myths, reflecting a more accurate and less mythicized view of love following intervention. Although we know of no prevention programs that have directly addressed myths about romantic love, previous studies have associated these myths with the origin and maintenance of dating violence [70,71]. Modifying these beliefs and myths could spell a substantial change in how adolescents deal with couple relationships, in such a way that potential violent behavior would no longer be justified by mistaken beliefs that mask love through control and dependence. Future follow-up measures will enable the analysis of how the modification of these beliefs influences the program’s impact on violence.

Regarding anger regulation and self-esteem, the program had a clear impact on self-esteem and showed a trend for anger regulation. Specifically, better anger regulation and decreased self-deprecation were observed in the experimental group participants following intervention. Although these results should be taken with caution, the findings are particularly noteworthy; they confirm that the program impacts positively on adolescents’ emotional competence, given that the negative views they hold of themselves decrease after intervention and their capacity to regulate their negative emotional states increases. Both anger regulation [72, 73] and self-esteem [74, 75] have been described extensively in literature as risk factors directly linked to dating violence, highlighting how partner aggression would arise as a result of difficulties in managing and controlling negative emotions, such as anger and annoyance, during an argument or conflict. Although we have not analyzed whether anger may have played a mediating role in greater or lesser dating violence reduction, a better handling and regulation of anger could lead to a more positive self-view and, consequently, a decrease in violence. Along these lines, Fellmeth et al. [15] recommended the need to work on adolescents’ self-esteem, especially among those exhibiting low self-esteem, as a way of increasing program efficacy. Future editions of the program could explore this.

Lastly, the fidelity analysis yielded reasonably positive results owing to the fact that, as the lessons progressed, the implementers worked toward complying with 100% of the content. Because fidelity was not 100% in the first lessons, this could indicate the need to adapt the content for these earlier sessions to ensure that all planned activities are fully implemented; it could also be indicative of the program implementers’ lack of knowledge about the group and how to handle it, meaning that they would end up spending more time on management tasks and class dynamic organization and less time on program activities. Fidelity to the program and to the implementation conditions at all development stages of an evidence-based intervention is essential [76], especially during the early trials of intervention programs which ensures good control over the variables that may affect implementation conditions [77]. Although 100% fidelity to the program has not been achieved, the data obtained may be considered acceptable given the complexity involved in controlling all variables at play in the daily class dynamic during the program implementation period.

To summarize, the evaluation of the first edition of the *Dat-e Adolescence* program showed that while it was effective at reducing factors directly related to violence, it did not directly impact on violence reduction. These results, although promising, should be interpreted with caution given that the program has only undergone one trial and no follow-up measures have been taken as yet. Future waves will allow us to evaluate whether the impact holds in the studied variables. Furthermore, the fact that the program has proved effective in terms of the predictors of dating violence considered in the program in accordance with the Dynamic Developmental Systems Model [36], suggests the need to undertake future analyses on the possible mediating effects of these variables on dating violence. In this regard, Gottfredson et al. [54] have emphasized the need to test the conceptual models [78] within interventions in order to test how the mediator variables relate to the outcomes, in this case dating violence. Future studies will therefore move forward in this direction.

### Limitations

Despite yielding promising results, this first edition of the *Dat-e Adolescence* program has some limitations worth noting. The first lies in the intervention’s design. Although the schools were randomly assigned to the control and experimental groups, it was the school staff who decided which classes would take part. Future editions benefiting from completely randomized trials would enable us to confirm whether the program’s efficacy is maintained. In the same vein, the program was implemented by researchers during this initial trial in line with recommendations made by Flay et al. [77] and Gottfredson et al. [54]. These same implementation conditions have been carried out in earlier programs [79, 80, among other], because it ensures greater control of the implementation conditions. However, teaching staff’s non-active participation in the program’s implementation could limit the potential impact they have on changing the school climate and culture when it comes to dating violence. Future editions would do well to consider teachers’ previous training as well as training in program implementation in order to test whether the program’s efficacy is maintained under more natural implementation conditions.

Another important limitation has to do with experimental mortality. Participant attrition was reported at around 25%, the main cause of experimental mortality being the students’ absence at the time of data collection. In this regard, and as noted in literature [16], it is essential that these experimental mortality rates are successfully lowered when implementing intervention-based programs with a view to reducing bias when evaluating efficacy. In this study we were able to minimize the chances of introducing bias in the results thanks to the use of the FIML method and model estimation in participants with and without attrition, obtaining the same outcomes. Nonetheless, future editions should seek to improve implementation and follow-up conditions in order to reduce cases lost. From this perspective, the period during which program implementation took place made it difficult to collect data in the second wave, given that the data collection times coincided with the end of term and the students’ end-of-term excursions.

With regard to generalizing the results, this research was carried out in two cities in the Andalucía region and at state high schools with a medium economic, social and cultural level, meaning that the results are generalizable to a population bearing similar characteristics. Future trials should be conducted among populations with different economic, social and cultural characteristics in order to test the program’s efficacy in other groups and sociocultural contexts.

Lastly, intervention fidelity was reported solely by the implementer. Future studies would benefit from including more than one trained observer and reporting interrater agreement. Similarly, it would be interesting to incorporate a student satisfaction measure and to analyze it complementary to the implementer’s observations with the aim of being able to change and improve the program content.

## Conclusions

This research presents the first evaluation of *Dat-e Adolescence*, a dating violence prevention program implemented in state high schools in the autonomous region of Andalucía, Spain. Given that programs developed in the country to date have been scare and are of low methodological quality, this program, albeit with some limitations, represents one of the first efficacy evaluations in line with standards of evidence carried out in Spain. The outcomes showed significant changes in beliefs about romantic love, self-esteem and the expected trend in emotion regulation, but there was no impact on modifying aggressive behavior, victimization and couple quality. While the results are promising, they do demand follow-up measures and new trials.
